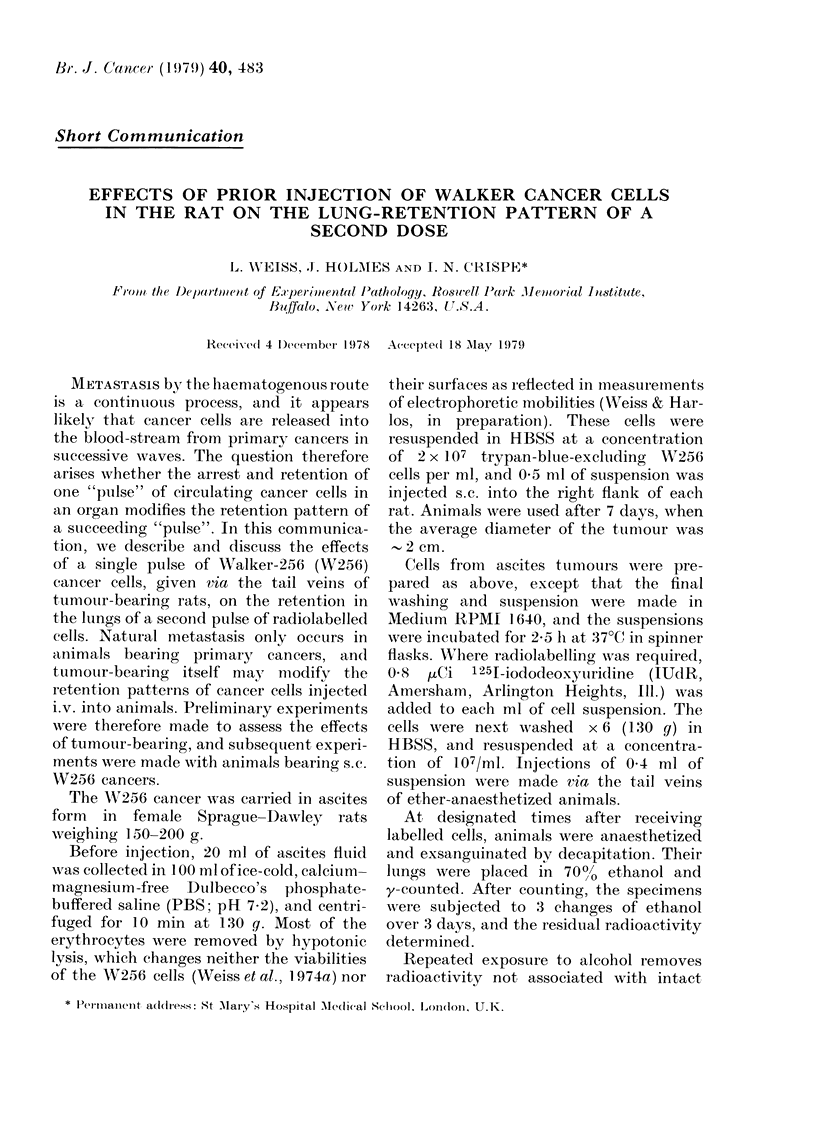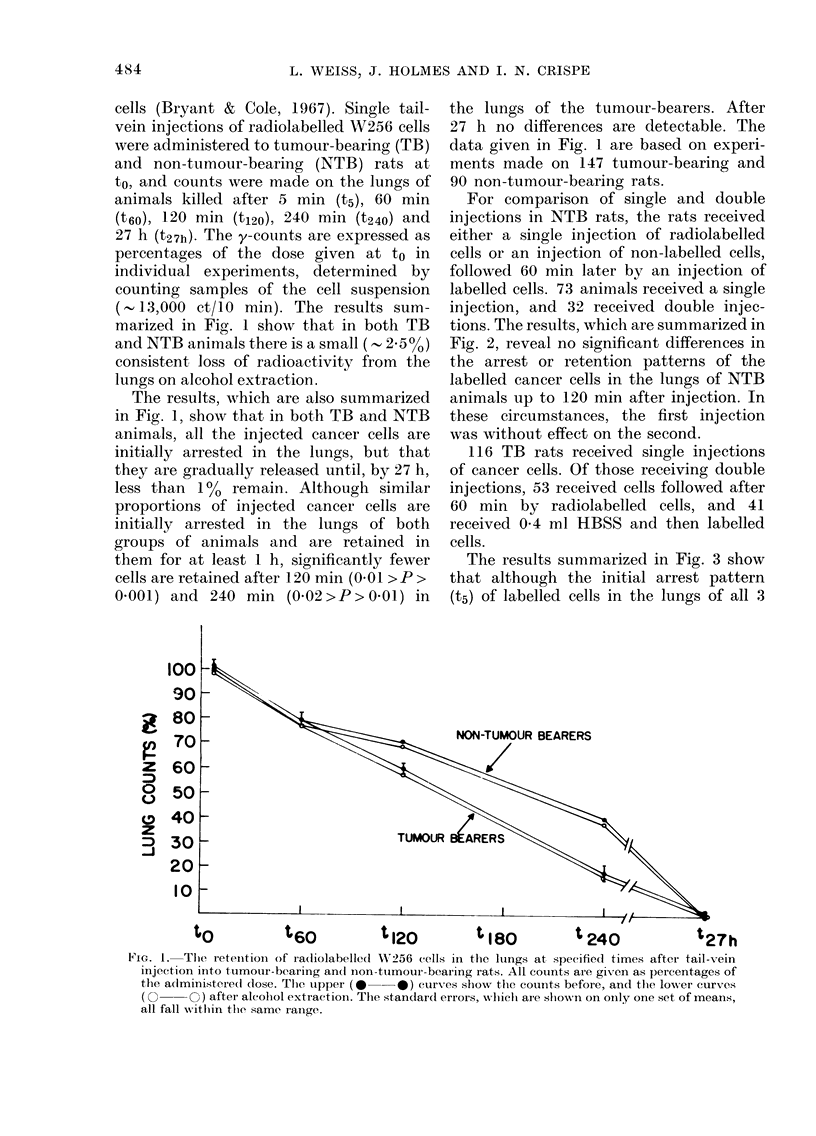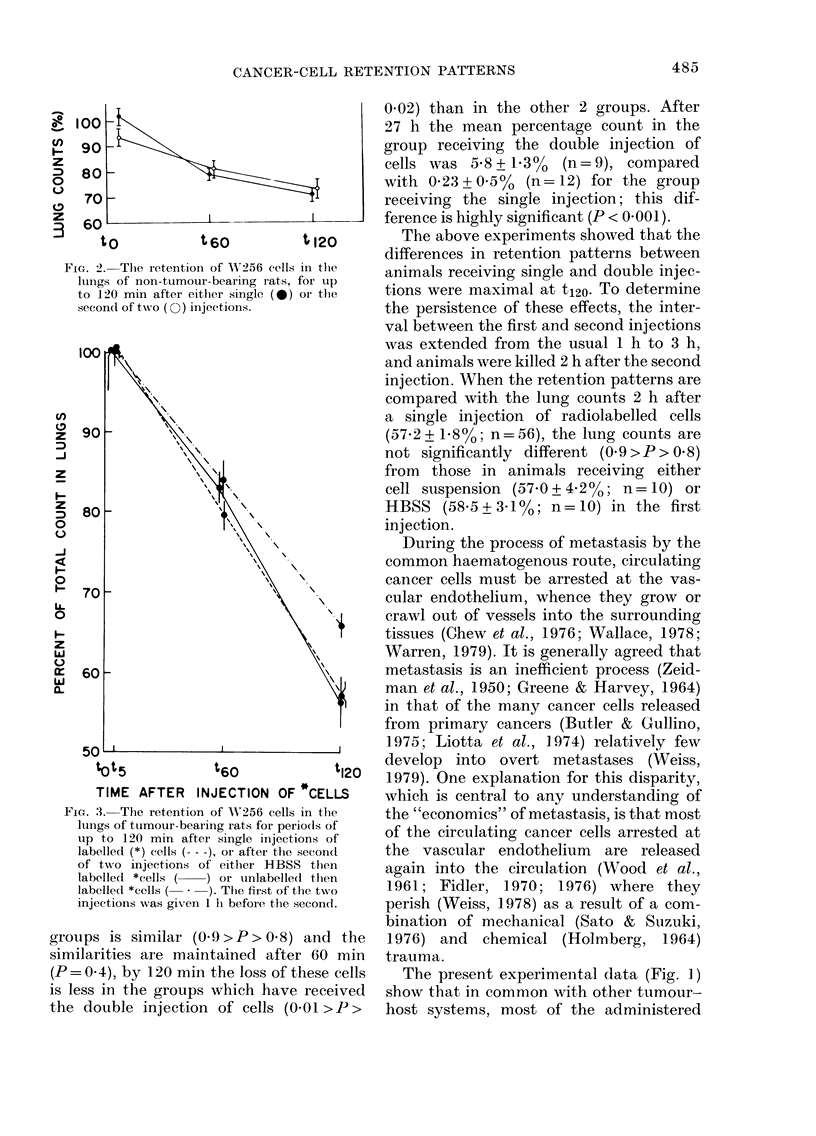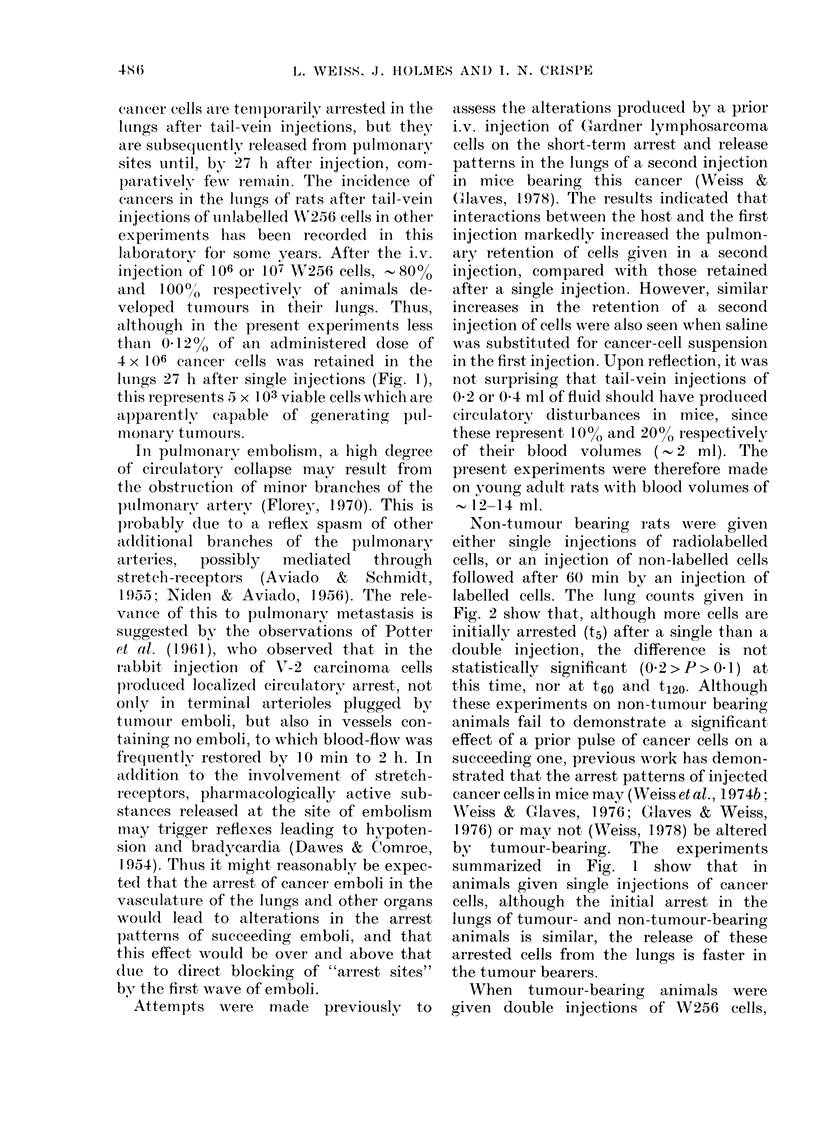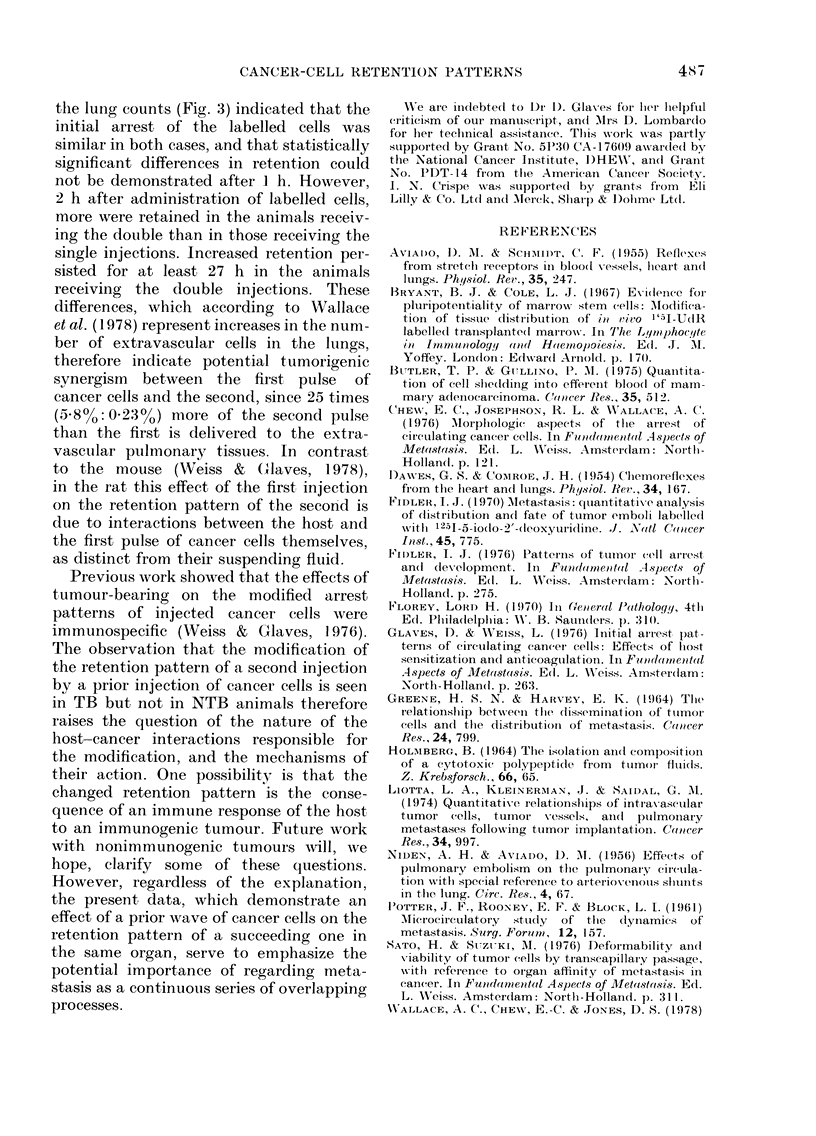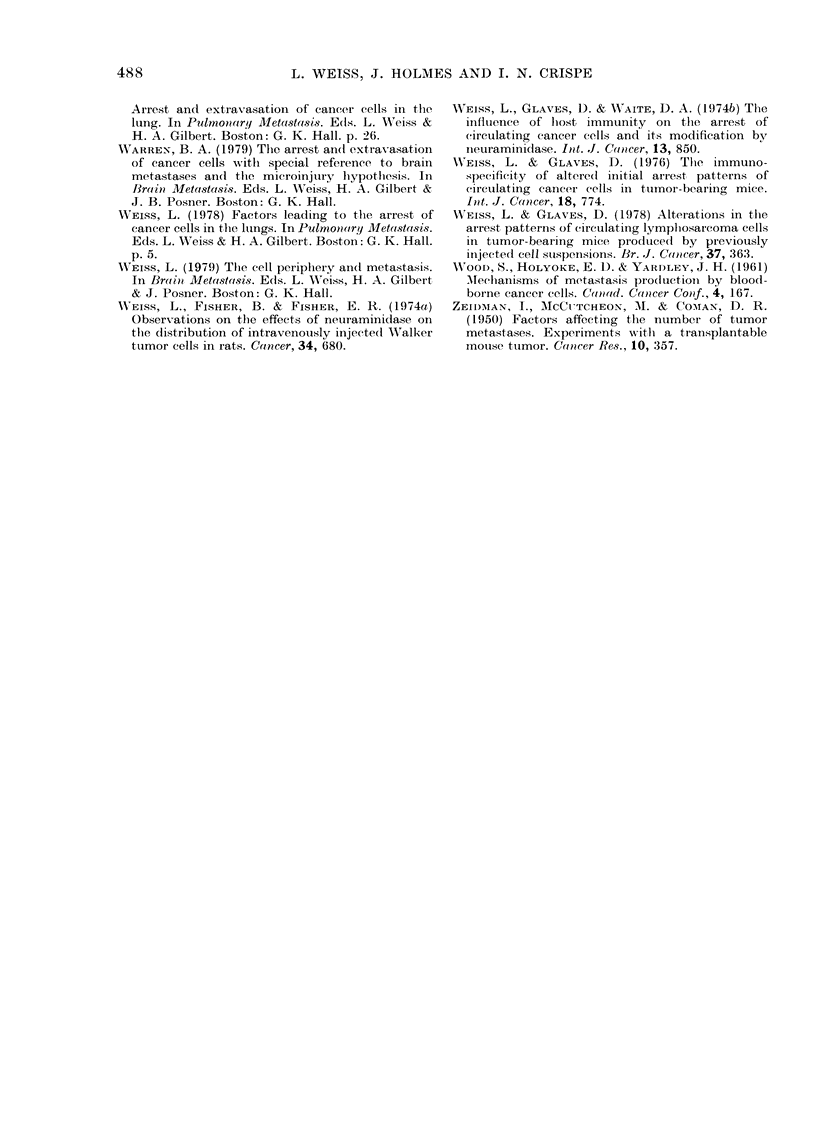# Effects of prior injection of Walker cancer cells in the rat on the lung-retention pattern of a second dose.

**DOI:** 10.1038/bjc.1979.204

**Published:** 1979-09

**Authors:** L. Weiss, J. Holmes, I. N. Crispe


*Br. J. Cancer* (1979) **40**, 483

***Short Communication***

### EFFECTS OF PRIOR INJECTION OF WALKER CANCER CELLS IN THE RAT ON THE LUNG-RETENTION PATTERN OF A SECOND DOSE

L. WEISS, J. HOLMES AND I. N. CRISPE*

*From the Department of Experimental Pathology, Roswell Park Memorial Institute, Buffalo, New York* 14263, *U.S.A.*

Received 4 December 1978 Accepted 18 May 1979

METASTASIS by the haematogenous route is a continuous process, and it appears likely that cancer cells are released into the blood-stream from primary cancers in successive waves. The question therefore arises whether the arrest and retention of one "pulse" of circulating cancer cells in an organ modifies the retention pattern of a succeeding "pulse". In this communication, we describe and discuss the effects of a single pulse of Walker-256 (W256) cancer cells, given *via* the tail veins of tumour-bearing rats, on the retention in the lungs of a second pulse of radiolabelled cells. Natural metastasis only occurs in animals bearing primary cancers, and tumour-bearing itself may modify the retention patterns of cancer cells injected i.v. into animals. Preliminary experiments were therefore made to assess the effects of tumour-bearing, and subsequent experiments were made with animals bearing s.c. W256 cancers.

The W256 cancer was carried in ascites form in female Sprague–Dawley rats weighing 150–200 g.

Before injection, 20 ml of ascites fluid was collected in 100 ml of ice-cold, calcium–magnesium-free Dulbecco's phosphate-buffered saline (PBS; pH 7·2), and centrifuged for 10 min at 130 *g*. Most of the erythrocytes were removed by hypotonic lysis, which changes neither the viabilities of the W256 cells (Weiss *et al.*, 1974*a*) nor their surfaces as reflected in measurements of electrophoretic mobilities (Weiss & Harlos, in preparation). These cells were resuspended in HBSS at a concentration of $2 \times 10^7$ trypan-blue-excluding W256 cells per ml, and 0·5 ml of suspension was injected s.c. into the right flank of each rat. Animals were used after 7 days, when the average diameter of the tumour was ~ 2 cm.

Cells from ascites tumours were prepared as above, except that the final washing and suspension were made in Medium RPMI 1640, and the suspensions were incubated for 2·5 h at 37°C in spinner flasks. Where radiolabelling was required, 0·8 $\mu$Ci $^{125}$I-iododeoxyuridine (IUdR, Amersham, Arlington Heights, Ill.) was added to each ml of cell suspension. The cells were next washed $\times 6$ (130 *g*) in HBSS, and resuspended at a concentration of $10^7$/ml. Injections of 0·4 ml of suspension were made *via* the tail veins of ether-anaesthetized animals.

At designated times after receiving labelled cells, animals were anaesthetized and exsanguinated by decapitation. Their lungs were placed in 70% ethanol and $\gamma$-counted. After counting, the specimens were subjected to 3 changes of ethanol over 3 days, and the residual radioactivity determined.

Repeated exposure to alcohol removes radioactivity not associated with intact

* Permanent address: St Mary's Hospital Medical School, London, U.K.

cells (Bryant & Cole, 1967). Single tail-vein injections of radiolabelled W256 cells were administered to tumour-bearing (TB) and non-tumour-bearing (NTB) rats at $t_0$, and counts were made on the lungs of animals killed after 5 min ($t_5$), 60 min ($t_{60}$), 120 min ($t_{120}$), 240 min ($t_{240}$) and 27 h ($t_{27h}$). The $\gamma$-counts are expressed as percentages of the dose given at $t_0$ in individual experiments, determined by counting samples of the cell suspension ($\sim$13,000 ct/10 min). The results summarized in Fig. 1 show that in both TB and NTB animals there is a small ($\sim$2·5%) consistent loss of radioactivity from the lungs on alcohol extraction.

The results, which are also summarized in Fig. 1, show that in both TB and NTB animals, all the injected cancer cells are initially arrested in the lungs, but that they are gradually released until, by 27 h, less than 1% remain. Although similar proportions of injected cancer cells are initially arrested in the lungs of both groups of animals and are retained in them for at least 1 h, significantly fewer cells are retained after 120 min ($0{\cdot}01 > P > 0{\cdot}001$) and 240 min ($0{\cdot}02 > P > 0{\cdot}01$) in the lungs of the tumour-bearers. After 27 h no differences are detectable. The data given in Fig. 1 are based on experiments made on 147 tumour-bearing and 90 non-tumour-bearing rats.

For comparison of single and double injections in NTB rats, the rats received either a single injection of radiolabelled cells or an injection of non-labelled cells, followed 60 min later by an injection of labelled cells. 73 animals received a single injection, and 32 received double injections. The results, which are summarized in Fig. 2, reveal no significant differences in the arrest or retention patterns of the labelled cancer cells in the lungs of NTB animals up to 120 min after injection. In these circumstances, the first injection was without effect on the second.

116 TB rats received single injections of cancer cells. Of those receiving double injections, 53 received cells followed after 60 min by radiolabelled cells, and 41 received 0·4 ml HBSS and then labelled cells.

The results summarized in Fig. 3 show that although the initial arrest pattern ($t_5$) of labelled cells in the lungs of all 3

FIG. 1.—The retention of radiolabelled W256 cells in the lungs at specified times after tail-vein injection into tumour-bearing and non-tumour-bearing rats. All counts are given as percentages of the administered dose. The upper (●——●) curves show the counts before, and the lower curves (○——○) after alcohol extraction. The standard errors, which are shown on only one set of means, all fall within the same range.

FIG. 2.—The retention of W256 cells in the lungs of non-tumour-bearing rats, for up to 120 min after either single (●) or the second of two (○) injections.

FIG. 3.—The retention of W256 cells in the lungs of tumour-bearing rats for periods of up to 120 min after single injections of labelled (*) cells (- - -), or after the second of two injections of either HBSS then labelled *cells (——) or unlabelled then labelled *cells (— · —). The first of the two injections was given 1 h before the second.

groups is similar ($0.9 > P > 0.8$) and the similarities are maintained after 60 min ($P = 0.4$), by 120 min the loss of these cells is less in the groups which have received the double injection of cells ($0.01 > P > 0.02$) than in the other 2 groups. After 27 h the mean percentage count in the group receiving the double injection of cells was $5.8 \pm 1.3\%$ (n = 9), compared with $0.23 \pm 0.5\%$ (n = 12) for the group receiving the single injection; this difference is highly significant ($P < 0.001$).

The above experiments showed that the differences in retention patterns between animals receiving single and double injections were maximal at $t_{120}$. To determine the persistence of these effects, the interval between the first and second injections was extended from the usual 1 h to 3 h, and animals were killed 2 h after the second injection. When the retention patterns are compared with the lung counts 2 h after a single injection of radiolabelled cells ($57.2 \pm 1.8\%$; n = 56), the lung counts are not significantly different ($0.9 > P > 0.8$) from those in animals receiving either cell suspension ($57.0 \pm 4.2\%$; n = 10) or HBSS ($58.5 \pm 3.1\%$; n = 10) in the first injection.

During the process of metastasis by the common haematogenous route, circulating cancer cells must be arrested at the vascular endothelium, whence they grow or crawl out of vessels into the surrounding tissues (Chew *et al.*, 1976; Wallace, 1978; Warren, 1979). It is generally agreed that metastasis is an inefficient process (Zeidman *et al.*, 1950; Greene & Harvey, 1964) in that of the many cancer cells released from primary cancers (Butler & Gullino, 1975; Liotta *et al.*, 1974) relatively few develop into overt metastases (Weiss, 1979). One explanation for this disparity, which is central to any understanding of the "economics" of metastasis, is that most of the circulating cancer cells arrested at the vascular endothelium are released again into the circulation (Wood *et al.*, 1961; Fidler, 1970; 1976) where they perish (Weiss, 1978) as a result of a combination of mechanical (Sato & Suzuki, 1976) and chemical (Holmberg, 1964) trauma.

The present experimental data (Fig. 1) show that in common with other tumour–host systems, most of the administered cancer cells are temporarily arrested in the lungs after tail-vein injections, but they are subsequently released from pulmonary sites until, by 27 h after injection, comparatively few remain. The incidence of cancers in the lungs of rats after tail-vein injections of unlabelled W256 cells in other experiments has been recorded in this laboratory for some years. After the i.v. injection of $10^6$ or $10^7$ W256 cells, $\sim 80\%$ and $100\%$ respectively of animals developed tumours in their lungs. Thus, although in the present experiments less than $0{\cdot}12\%$ of an administered dose of $4 \times 10^6$ cancer cells was retained in the lungs 27 h after single injections (Fig. 1), this represents $5 \times 10^3$ viable cells which are apparently capable of generating pulmonary tumours.

In pulmonary embolism, a high degree of circulatory collapse may result from the obstruction of minor branches of the pulmonary artery (Florey, 1970). This is probably due to a reflex spasm of other additional branches of the pulmonary arteries, possibly mediated through stretch-receptors (Aviado & Schmidt, 1955; Niden & Aviado, 1956). The relevance of this to pulmonary metastasis is suggested by the observations of Potter *et al.* (1961), who observed that in the rabbit injection of V-2 carcinoma cells produced localized circulatory arrest, not only in terminal arterioles plugged by tumour emboli, but also in vessels containing no emboli, to which blood-flow was frequently restored by 10 min to 2 h. In addition to the involvement of stretch-receptors, pharmacologically active substances released at the site of embolism may trigger reflexes leading to hypotension and bradycardia (Dawes & Comroe, 1954). Thus it might reasonably be expected that the arrest of cancer emboli in the vasculature of the lungs and other organs would lead to alterations in the arrest patterns of succeeding emboli, and that this effect would be over and above that due to direct blocking of "arrest sites" by the first wave of emboli.

Attempts were made previously to assess the alterations produced by a prior i.v. injection of Gardner lymphosarcoma cells on the short-term arrest and release patterns in the lungs of a second injection in mice bearing this cancer (Weiss & Glaves, 1978). The results indicated that interactions between the host and the first injection markedly increased the pulmonary retention of cells given in a second injection, compared with those retained after a single injection. However, similar increases in the retention of a second injection of cells were also seen when saline was substituted for cancer-cell suspension in the first injection. Upon reflection, it was not surprising that tail-vein injections of 0·2 or 0·4 ml of fluid should have produced circulatory disturbances in mice, since these represent $10\%$ and $20\%$ respectively of their blood volumes ($\sim 2$ ml). The present experiments were therefore made on young adult rats with blood volumes of $\sim 12$–14 ml.

Non-tumour bearing rats were given either single injections of radiolabelled cells, or an injection of non-labelled cells followed after 60 min by an injection of labelled cells. The lung counts given in Fig. 2 show that, although more cells are initially arrested ($t_5$) after a single than a double injection, the difference is not statistically significant ($0{\cdot}2 > P > 0{\cdot}1$) at this time, nor at $t_{60}$ and $t_{120}$. Although these experiments on non-tumour bearing animals fail to demonstrate a significant effect of a prior pulse of cancer cells on a succeeding one, previous work has demonstrated that the arrest patterns of injected cancer cells in mice may (Weiss *et al.*, 1974*b*; Weiss & Glaves, 1976; Glaves & Weiss, 1976) or may not (Weiss, 1978) be altered by tumour-bearing. The experiments summarized in Fig. 1 show that in animals given single injections of cancer cells, although the initial arrest in the lungs of tumour- and non-tumour-bearing animals is similar, the release of these arrested cells from the lungs is faster in the tumour bearers.

When tumour-bearing animals were given double injections of W256 cells, the lung counts (Fig. 3) indicated that the initial arrest of the labelled cells was similar in both cases, and that statistically significant differences in retention could not be demonstrated after 1 h. However, 2 h after administration of labelled cells, more were retained in the animals receiving the double than in those receiving the single injections. Increased retention persisted for at least 27 h in the animals receiving the double injections. These differences, which according to Wallace *et al.* (1978) represent increases in the number of extravascular cells in the lungs, therefore indicate potential tumorigenic synergism between the first pulse of cancer cells and the second, since 25 times (5·8%: 0·23%) more of the second pulse than the first is delivered to the extravascular pulmonary tissues. In contrast to the mouse (Weiss & Glaves, 1978), in the rat this effect of the first injection on the retention pattern of the second is due to interactions between the host and the first pulse of cancer cells themselves, as distinct from their suspending fluid.

Previous work showed that the effects of tumour-bearing on the modified arrest patterns of injected cancer cells were immunospecific (Weiss & Glaves, 1976). The observation that the modification of the retention pattern of a second injection by a prior injection of cancer cells is seen in TB but not in NTB animals therefore raises the question of the nature of the host–cancer interactions responsible for the modification, and the mechanisms of their action. One possibility is that the changed retention pattern is the consequence of an immune response of the host to an immunogenic tumour. Future work with nonimmunogenic tumours will, we hope, clarify some of these questions. However, regardless of the explanation, the present data, which demonstrate an effect of a prior wave of cancer cells on the retention pattern of a succeeding one in the same organ, serve to emphasize the potential importance of regarding metastasis as a continuous series of overlapping processes.

We are indebted to Dr D. Glaves for her helpful criticism of our manuscript, and Mrs D. Lombardo for her technical assistance. This work was partly supported by Grant No. 5P30 CA-17609 awarded by the National Cancer Institute, DHEW, and Grant No. PDT-14 from the American Cancer Society. I. N. Crispe was supported by grants from Eli Lilly & Co. Ltd and Merck, Sharp & Dohme Ltd.

### REFERENCES

Aviado, D. M. & Schmidt, C. F. (1955) Reflexes from stretch receptors in blood vessels, heart and lungs. *Physiol. Rev.*, **35,** 247.

Bryant, B. J. & Cole, L. J. (1967) Evidence for pluripotentiality of marrow stem cells: Modification of tissue distribution of *in vivo* $^{125}$I-UdR labelled transplanted marrow. In *The Lymphocyte in Immunology and Haemopoiesis.* Ed. J. M. Yoffey. London: Edward Arnold. p. 170.

Butler, T. P. & Gullino, P. M. (1975) Quantitation of cell shedding into efferent blood of mammary adenocarcinoma. *Cancer Res.*, **35,** 512.

Chew, E. C., Josephson, R. L. & Wallace, A. C. (1976) Morphologic aspects of the arrest of circulating cancer cells. In *Fundamental Aspects of Metastasis.* Ed. L. Weiss. Amsterdam: North-Holland. p. 121.

Dawes, G. S. & Comroe, J. H. (1954) Chemoreflexes from the heart and lungs. *Physiol. Rev.*, **34,** 167.

Fidler, I. J. (1970) Metastasis: quantitative analysis of distribution and fate of tumor emboli labelled with $^{125}$I-5-iodo-2′-deoxyuridine. *J. Natl Cancer Inst.*, **45,** 775.

Fidler, I. J. (1976) Patterns of tumor cell arrest and development. In *Fundamental Aspects of Metastasis.* Ed. L. Weiss. Amsterdam: North-Holland. p. 275.

Florey, Lord H. (1970) In *General Pathology*, 4th Ed. Philadelphia: W. B. Saunders. p. 310.

Glaves, D. & Weiss, L. (1976) Initial arrest patterns of circulating cancer cells: Effects of host sensitization and anticoagulation. In *Fundamental Aspects of Metastasis.* Ed. L. Weiss. Amsterdam: North-Holland. p. 263.

Greene, H. S. N. & Harvey, E. K. (1964) The relationship between the dissemination of tumor cells and the distribution of metastasis. *Cancer Res.*, **24,** 799.

Holmberg, B. (1964) The isolation and composition of a cytotoxic polypeptide from tumor fluids. *Z. Krebsforsch.*, **66,** 65.

Liotta, L. A., Kleinerman, J. & Saidal, G. M. (1974) Quantitative relationships of intravascular tumor cells, tumor vessels, and pulmonary metastases following tumor implantation. *Cancer Res.*, **34,** 997.

Niden, A. H. & Aviado, D. M. (1956) Effects of pulmonary embolism on the pulmonary circulation with special reference to arteriovenous shunts in the lung. *Circ. Res.*, **4,** 67.

Potter, J. F., Rooney, E. F. & Block, L. I. (1961) Microcirculatory study of the dynamics of metastasis. *Surg. Forum*, **12,** 157.

Sato, H. & Suzuki, M. (1976) Deformability and viability of tumor cells by transcapillary passage, with reference to organ affinity of metastasis in cancer. In *Fundamental Aspects of Metastasis.* Ed. L. Weiss. Amsterdam: North-Holland. p. 311.

Wallace, A. C., Chew, E.-C. & Jones, D. S. (1978)

Arrest and extravasation of cancer cells in the lung. In *Pulmonary Metastasis*. Eds. L. Weiss & H. A. Gilbert. Boston: G. K. Hall. p. 26.

WARREN, B. A. (1979) The arrest and extravasation of cancer cells with special reference to brain metastases and the microinjury hypothesis. In *Brain Metastasis*. Eds. L. Weiss, H. A. Gilbert & J. B. Posner. Boston: G. K. Hall.

WEISS, L. (1978) Factors leading to the arrest of cancer cells in the lungs. In *Pulmonary Metastasis*. Eds. L. Weiss & H. A. Gilbert. Boston: G. K. Hall. p. 5.

WEISS, L. (1979) The cell periphery and metastasis. In *Brain Metastasis*. Eds. L. Weiss, H. A. Gilbert & J. Posner. Boston: G. K. Hall.

WEISS, L., FISHER, B. & FISHER, E. R. (1974*a*) Observations on the effects of neuraminidase on the distribution of intravenously injected Walker tumor cells in rats. *Cancer*, **34**, 680.

WEISS, L., GLAVES, D. & WAITE, D. A. (1974*b*) The influence of host immunity on the arrest of circulating cancer cells and its modification by neuraminidase. *Int. J. Cancer*, **13**, 850.

WEISS, L. & GLAVES, D. (1976) The immunospecificity of altered initial arrest patterns of circulating cancer cells in tumor-bearing mice. *Int. J. Cancer*, **18**, 774.

WEISS, L. & GLAVES, D. (1978) Alterations in the arrest patterns of circulating lymphosarcoma cells in tumor-bearing mice produced by previously injected cell suspensions. *Br. J. Cancer*, **37**, 363.

WOOD, S., HOLYOKE, E. D. & YARDLEY, J. H. (1961) Mechanisms of metastasis production by blood-borne cancer cells. *Canad. Cancer Conf.*, **4**, 167.

ZEIDMAN, I., McCUTCHEON, M. & COMAN, D. R. (1950) Factors affecting the number of tumor metastases. Experiments with a transplantable mouse tumor. *Cancer Res.*, **10**, 357.